# Dual deep learning approach for non-invasive renal tumour subtyping with VERDICT-MRI

**DOI:** 10.1038/s44303-025-00135-6

**Published:** 2026-01-06

**Authors:** Snigdha Sen, Lorna Smith, Lucy Caselton, Joey Clemente, Maxine Tran, Shonit Punwani, David Atkinson, Richard L. Hesketh, Eleftheria Panagiotaki

**Affiliations:** 1 UCL Hawkes Institute, 90 High Holborn, London, UK; 2 UCL Centre for Medical Imaging, Charles Bell House, London, UK; 3 UCL Advanced Research Computing Centre, 90 High Holborn, London, UK; 4 UCL Division of Surgery and Interventional Science, London, UK; 5 UCL Department of Imaging, Charles Bell House, London, UK

**Keywords:** Cancer, Medical research, Oncology, Urology

## Abstract

Renal cell carcinomas (RCCs) have multiple subtypes that are difficult to distinguish using imaging alone. This study characterises renal tumour microstructure using diffusion MRI (dMRI) and the Vascular, Extracellular and Restricted Diffusion for Cytometry in Tumours (VERDICT)-MRI framework. Patients were prospectively recruited from the RIM trial (ClinicalTrials.gov: NCT07173140, 20/11/2024). Fourteen patients with 17 renal tumours (including benign and various RCC subtypes) underwent dMRI using nine *b*-values (0–2500 s/mm²). A three-compartment VERDICT model was fitted with a self-supervised neural network. Compared to simpler dMRI models, VERDICT more accurately captured the diffusion data in tumour and healthy tissue. VERDICT revealed significant differences in intracellular volume fraction between cancerous and normal tissue, and in vascular volume fraction between vascular and non-vascular regions. A feature selection method identified a reduced 4 *b*-value protocol (b = [70, 150, 1000, 2000]), cutting scan time by over 30 min, enabling more efficient imaging in larger cohorts.

## Introduction

Renal cancer accounts for roughly 2% of all cancer diagnoses and deaths worldwide^1^. Over 90% of primary renal cancers are renal cell carcinoma (RCC), arising from the renal tubule epithelium^2^. The 2022 WHO classification subdivides RCC into 21 different histotypes of which the commonest are clear-cell RCC (ccRCC) (~70%), papillary RCC (pRCC) (~10%) and chromophobe (chRCC) (~5%)^3^. Benign tumours of the kidney can also occur, notably oncocytoma and angiomyolipoma^4^. The main diagnostic imaging for renal tumours is computed tomography (CT), which can diagnose RCC with a sensitivity of over 95% and a specificity of around 90%^5^. While different tumour subtypes exhibit typical imaging features on both CT and MRI, there is considerable overlap between certain types e.g., oncocytoma, clear cell and chRCC^6^. With the exception of the identification of “macroscopic fat” in lipid-rich angiomyolipomas, there are currently no imaging biomarkers with sufficient accuracy to differentiate benign and malignant tumours in clinical practice^7^. A historical reluctance to use biopsy due to the risks, sampling error and a potentially low negative predictive value have meant that rates of benign histology following nephrectomy remain as high as 31%^8^.

Therefore, new imaging biomarkers are required that are capable of accurately defining both lesion subtype and metastatic risk. MRI techniques offer valuable insights into tumour biology; particularly diffusion-weighted (DW) MRI, which can access restricted diffusion via the apparent diffusion coefficient (ADC). DW-MRI has been used to differentiate RCC subgroups^9–11^ and has proven useful in characterising indeterminate small renal lesions, whether inflammatory or malignant, as both may exhibit restricted diffusion^12^. However, whilst most clinical DW-MRI studies focus on the ADC, it conflates multiple physiological parameters, reducing its specificity for identifying disease mechanisms, with studies showing varying performance in RCC subtype characterisation^13–15^. The intravoxel incoherent motion (IVIM) model, which separates diffusion in tissue from pseudo-diffusion in blood vessels, has demonstrated enhanced performance by capturing tumour vascularity^16–18^, as flow compartments play a significant role in microstuctural imaging of the kidney^19^. While IVIM improves upon the uni-compartmental ADC, it still oversimplifies the complex underlying tissue microstructure.

The Vascular, Extracellular and Restricted Diffusion for Cytometry in Tumours (VERDICT)-MRI framework combines a specific diffusion acquisition protocol with a three-compartment biophysical model of the DW-MRI signal in tumour tissue^20^. Previously, VERDICT has shown effective tumour characterisation in colorectal^21^, prostate^22,23^ and brain tumours^24^. Specifically, in prostate cancer where the problem of lesion over-treatment is similar to that in renal tumours, VERDICT improves differentiation between benign and low-grade malignant lesions compared to the ADC^25,26^. In this work, we trial VERDICT-MRI for renal tumours for the first time, in a cohort of 14 patients with 17 renal masses. The patients participated in a specific DW acquisition to obtain rich datasets for modelling analysis, and a kidney-specific VERDICT model was identified and fitted to this data using a self-supervised deep learning method^27^. The acquisition of comprehensive datasets took 46 min; a clinically impractical acquisition time. Traditionally, optimised protocols are identified via the Fisher information, a computationally expensive and model-restricted approach^28^. We investigated recursive feature elimination methods that have been used previously to identify optimal VERDICT imaging protocols^29,30^, with a model-free approach adaptable to different tasks.

We found that our VERDICT model for renal tissue outperformed ADC and IVIM at describing the diffusion-weighted data and underlying microstructure. The VERDICT intracellular volume fraction was significantly higher in cancer than normal kidney tissue and revealed similar trends to those predicted by histology, whilst the vascular volume fraction was statistically higher in vascular tumours than in non-vascular. Finally, our dual-network feature selection method identified an economical acquisition protocol for clinical use, reducing the scan time by 32 min and maintaining diagnostic power comparable to the full protocol.

## Results

### Patient demographics

Fourteen patients with 17 tumours (15 of which had histological confirmation and were included in the analysis) underwent MRI examination. The patients had a mean age of 66 ± 11 years, and a 1:3.6 female:male ratio (Table 1).

**Table 1:** Tumour types included in the study

Tumour type	Total	WHO/ISUP grades
ccRCC	5	1 (n = 3), 2 (n = 2)
pRCC	2	1 (n = 1), 3 (n = 1)
Unclassified RCC (uRCC)	1	3 (n = 1)
chRCC^a^	4	n/a
Classical oncocytoma	3	n/a

^a^Includes low grade oncocytic neoplasm.

### Histological validation of the VERDICT model

**Fig. 1:**
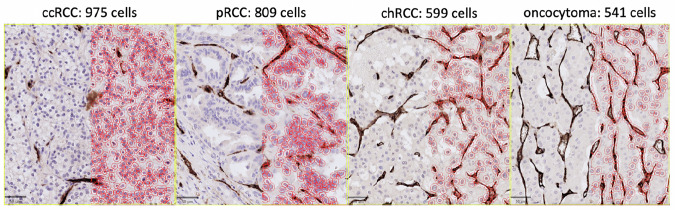
Histological analysis of cell size using H&E-staining. We observe that ccRCC and pRCC have a higher number of cells in the same area (180,000 mm^2^) of tumour in comparison to chRCC and oncocytoma, which agrees with the literature that they are more cellular tumour types.

ccRCC and pRCC demonstrated higher cell density in comparison to chRCC and oncocytoma (Fig. 1). Vascularity, measured by CD34 staining, grouped tumours into a vascular group of tumours (ccRCC, 12.6% positivity; oncocytoma, 10.4%; and chRCC, 7.1%) and non-vascular tumours (pRCC, 2.8%).

### VERDICT, IVIM and ADC model fitting of normal and cancerous tissue

**Fig. 2:**
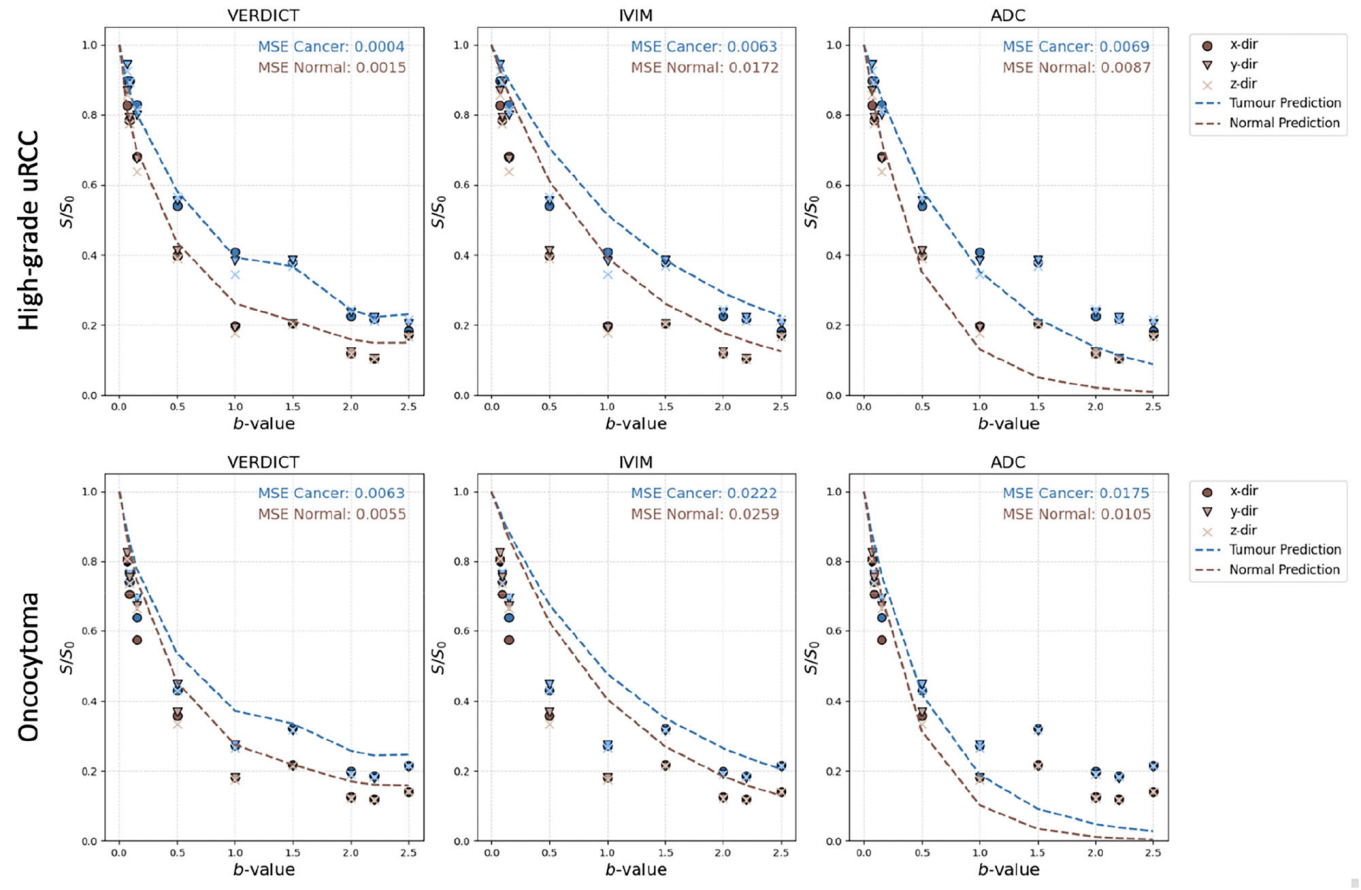
Signal prediction vs DWI data. Signal prediction vs DWI data for tumour and normal renal tissue via VERDICT vs. IVIM vs. ADC for a high-grade tumour and benign oncocytoma. The DW signal data are plotted as the points, the curve represents the model’s signal prediction and the MSE is calculated as the difference between the true and predicted value.

The VERDICT, IVIM and ADC models were fitted to the data from ROIs in tumour and normal kidney in Fig. 2. In both low and high-grade renal tumours, the mean-squared error (MSE) of the VERDICT fit is lower than ADC and IVIM in both normal and cancerous tissue, suggesting that it describes the data most accurately.

### VERDICT and ADC parameter estimates

**Fig. 3:**
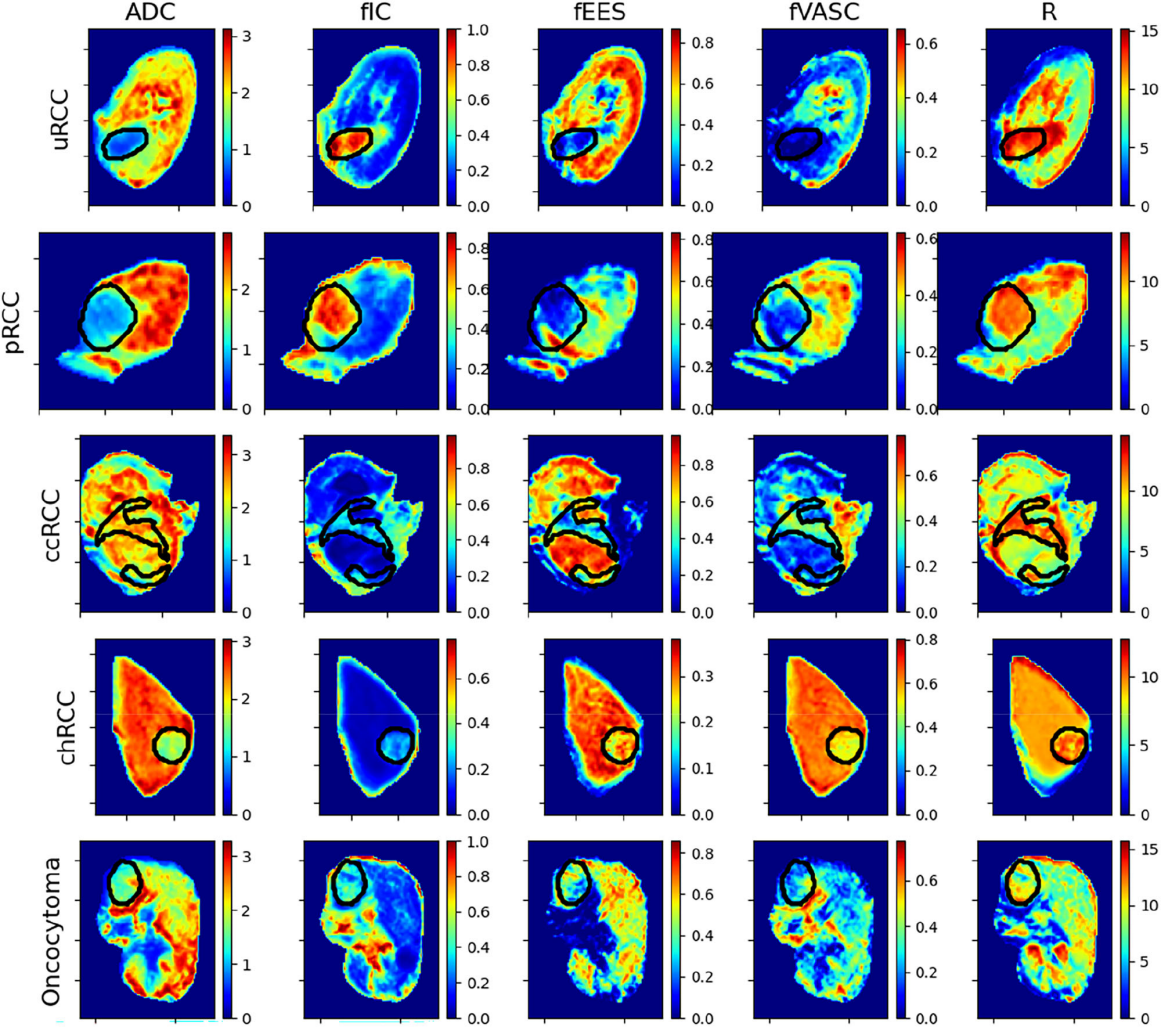
Parameter maps for ADC and VERDICT parameters in all patient groups. We observe higher and for more cellular tumours, (uRCC, pRCC and ccRCC), and elevated in more vascular masses (ccRCC, oncocytoma). ADC is lower for uRCC, pRCC and chRCC, but not for the other groups.

**Fig. 4:**
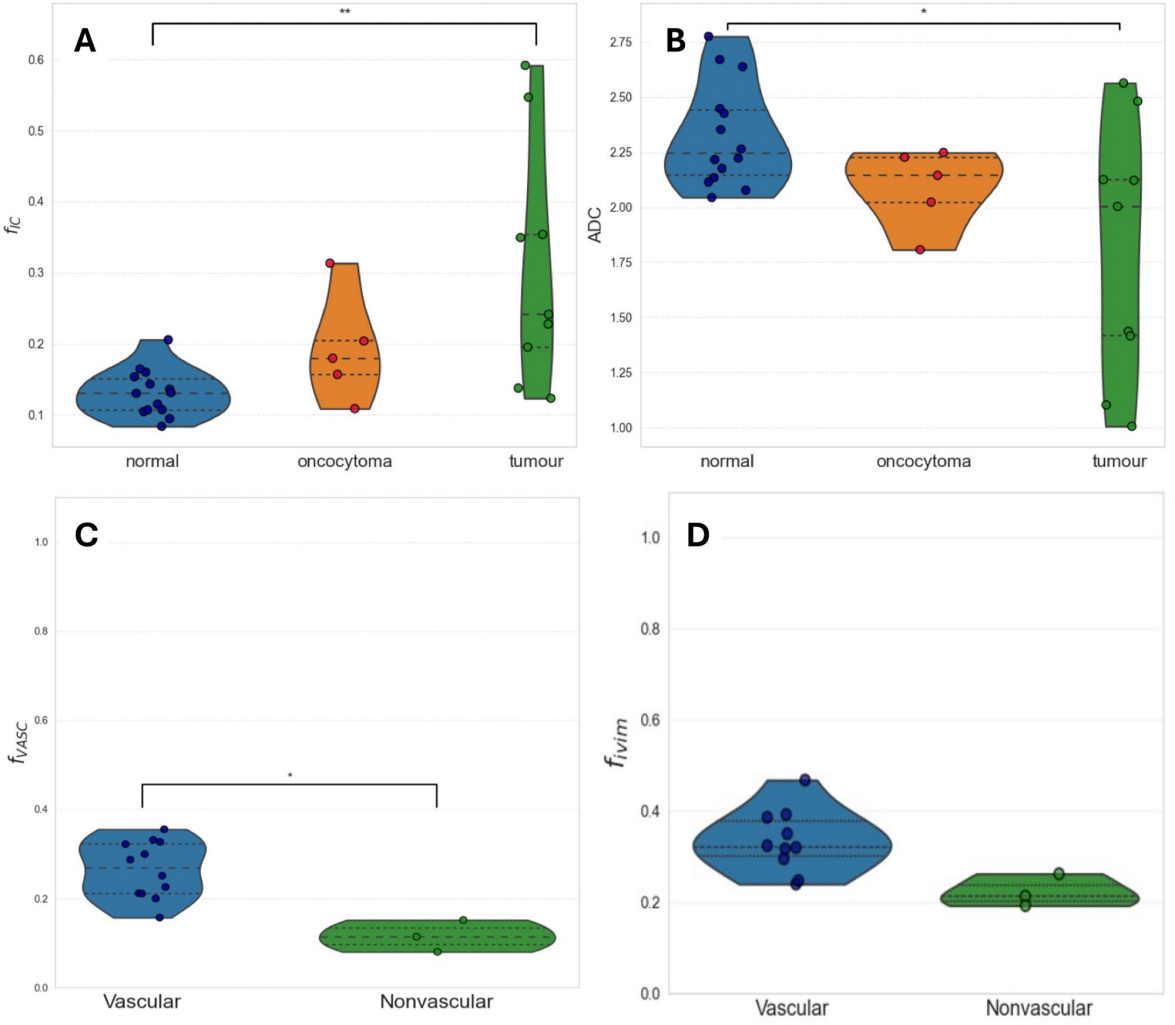
Parameter estimation for disease groups. **A** VERDICT f_IC_ and **B** ADC in normal kidney tissue, benign oncocytomas and cancerous tumours. The f_IC_ can discriminate cancer from normal tissue (*p* < 0.01), as can ADC with *p* < 0.05. **C** VERDICT f_VASC_ and **D** IVIM f_IVIM_ in vascular vs. non-vascular tumours – the f_VASC_ can discriminate these (*p* < 0.05).

**Fig. 5:**
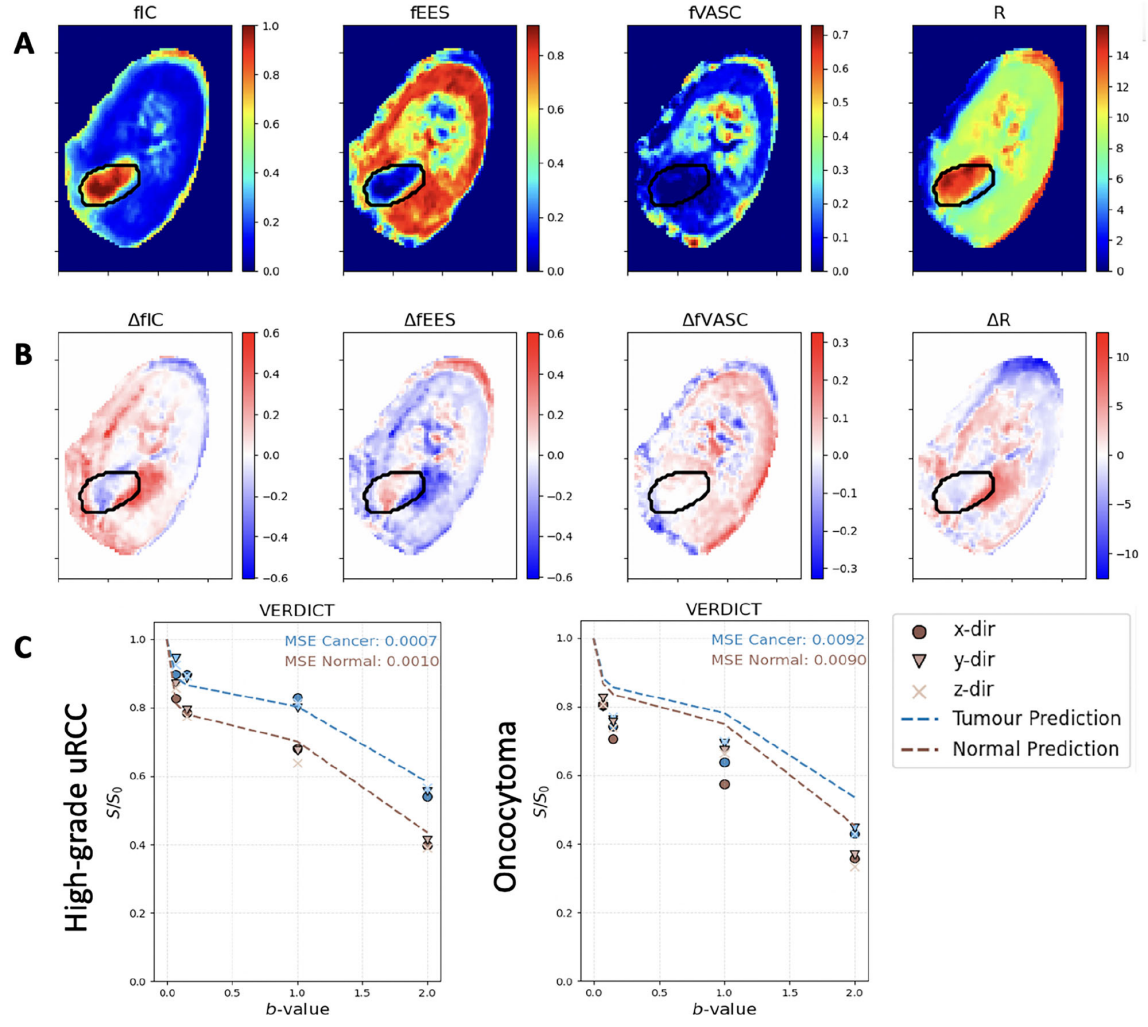
Protocol optimisation results. In (**A**) we show the maps produced using the identified shorter protocol, and in (**B**) we plot the difference between these maps and the original. We observe that they maintain the map features with some smoothing, and more variation is seen outside the tumour region. In (**C**) we plot the signal prediction vs. DWI data for a high-grade tumour and benign oncocytoma, observing that the MSE between the data and predictions remains similar to that seen with the full protocol.

Parametric maps were generated for the VERDICT parameters (intracellular volume fraction; ${f}_{{IC}}$, extracellular-extravascular volume fraction; ${f}_{{EES}}$, vascular volume fraction; ${f}_{{VASC}}$, cell radius; $R$) and ADC, for each of the patient groupings (Fig. 3). ${f}_{{IC}}$ and ADC ranged between 0.06 and 0.21 (standard deviation (SD): 0.03) and 2.05 and 2.76 (SD: 0.2), respectively, for benign renal parenchyma across all patients. ${f}_{{IC}}$ discriminated between cancerous from normal tissue (0.32 vs. 0.12; *p* < 0.01), as did ADC (1.6 vs. 2.3; *p* < 0.05) (Fig. 5a, b). The ${f}_{{IC}}$ for oncocytomas (benign) was not significantly different to normal tissue (0.17 vs. 0.12; *p* = n.s.). There were trends towards higher ${f}_{{IC}}$ and $R$ in the high-grade uRCC (${f}_{{IC}}$ = 0.59) and pRCC (${f}_{{IC}}$ = 0.55) compared to low grade tumours (${f}_{{IC}}$ = 0.21 ± 0.07) (Figs. 3 and 4). A similar trend was seen with ADC.

The tumours were grouped into vascular (ccRCC, oncocytoma and chromophobe tumours), and non-vascular (uRCC, pRCC) based on the unpaired histological analysis and degree of enhancement demonstrated on nephrographic-phase CT for each patient.

The ${f}_{{VASC}}$ was significantly higher in the vascular tumours than in non-vascular (mean ± SD: 0.29 ± 0.05 vs. 0.13 ± 0.02, *p* < 0.05; Fig. 5d), but there was no significance for $f$(0.33 ± 0.07 vs. 0.23 ± 0.03, *p* = n.s.; Fig. 5c).

### Deep learning protocol optimisation

The deep learning feature selection method produced a protocol of four optimally-informative *b*-values: 70, 150, 1000, and 2000 s/mm^2^. In Fig. 5, we reproduce our results with this protocol, demonstrating the quality of the parameter maps is largely preserved, with some smoothing. We plot the differences between these maps and the original and find that they are generally small with more variation outside of the tumour. We also find the MSE between the signal predictions and DW data remains similar to Fig. 2 in a benign and cancerous tumour. In Supplementary Fig. 3, we recreate Fig. 4 with the optimised protocol, finding similar statistical significance to the full protocol. The optimised protocol has an acquisition time of ~14 min, a reduction of over 30 min from the full protocol.

## Discussion

In this work, we create a model for renal tumours within the VERDICT-MRI framework for the first time, in a cohort of 14 patients. We acquire rich datasets of nine *b*-values to develop and fit a three-compartment VERDICT model for the kidney using self-supervised deep learning. We show that VERDICT provides a better description of the data than IVIM and ADC and obtain statistically significant differences between tissue types in the intracellular and vascular volume fractions. Finally, we find that our results hold when using a significantly shortened protocol of four *b*-values, demonstrating that this technique can be translated to the clinic with ease.

In Fig. 2, we show that the predicted signal from VERDICT matches the DW data more closely in terms of MSE than IVIM or ADC in both tumour and benign tissue. This suggests VERDICT provides a better description of the underlying microstructure, agreeing with previous studies where VERDICT outperforms both models in the prostate^20,23^. We note the MSE is lower in tumour regions than benign; we hypothesise that this is because the model is developed to describe the DW signal in tumour tissue specifically, therefore describes those features more accurately.

Histologically, ccRCC and pRCC demonstrated higher cellularity than chRCC and benign oncocytoma (Fig. 1), while chRCC and oncocytoma appear similar, explaining the challenge of differentiating these tumours on imaging. This also agrees with our results in Supplementary Fig. 2, where we note that for ${f}_{{IC}}$, ADC and $D$, our results overlap considerably for chRCC and oncocytoma. The higher ${f}_{{IC}}$ estimates in cancerous tumours, particularly the high-grade tumours, compared to the normal renal parenchyma agrees with prior knowledge that cancerous tumours are typically hypercellular^31^. Benign oncocytomas could not be discriminated from either normal tissue or cancer at a statistically significant level with either or ADC however, highlighting their confounding nature.

The unique microstructural information available via VERDICT compared to IVIM and ADC permitted comparison of tumour vascularity. Informed by our prior knowledge from CT imaging we grouped tumours into a vascular and non-vascular group based on their CT-enhancement characteristics and showed the ${f}_{{VASC}}$ to be significantly higher in the vascular tumours group. Inferring enhancement from DWI and VERDICT could potentially avoid contrast administration clinically, and further studies into diffusion time dependence could lead to the extraction of specific microstructural measurements^32^.

Lastly, in Fig. 5 we recreate our results with the optimised protocol identified via deep-learning-based feature selection and show consistency with those achieved with the full protocol. Along with Supplementary Fig. 3, these results demonstrate that similar diagnostic performance can be achieved whilst reducing the acquisition time by over 30 min, enabling the clinical translation of this work. This result also motivates the development of VERDICT models for other organs, as we demonstrate how the use of a few comprehensive datasets for model development can be used to identify clinical acquisitions.

This work is limited primarily by the small patient cohort, resulting in few patients per tumour subtype, which limits the statistical significance of these results. This is in part due to the long acquisition protocol; with the identified economical protocol, we hope to soon be able to trial VERDICT on a much larger number of kidney patients with a variety of subtypes. Additionally, whilst some histological analysis has been conducted, these are not paired samples with the imaging data, meaning that the imaging biomarkers cannot be fully validated histologically. Future studies will include paired histology and DWI for more in-depth analysis, such as quantitative correlations between fIC and cellularity and fVASC and CD34-based vascularity. We also note some field inhomogeneities in the images due to the EPI sequence used; future work will investigate distortion correction methods^33^. Finally, further information about vascularity could be extracted via the separation of vascularity into two distinct contributors – perfusion and tubular flow – or incorporation of spatial profiling of the renal parenchyma^34–37^. Such studies propose significant extensions to our current model, therefore we leave this avenue for future work.

In conclusion, this work develops a VERDICT-MRI model for renal tumours, and analyses results in a cohort of 14 patients. We show that VERDICT provides better data description and enhanced microstructural information over ADC, such as differences between patient groups in intracellular and vascular volume fractions. We exploit novel deep learning advances for both the model fitting approach to ensure robustness, and to identify a significantly shorter acquisition protocol. We find that we can extract equivalent diagnostic performance with our abbreviated protocol as with the original, allowing for smooth clinical translation of the technique.

## Methods

### Patient cohort

The RIM study (ClinicalTrials.gov: NCT07173140, 20/11/2024) was approved by the UCL Research Ethics Committee (07/Q0502/15) and all patients provided informed written consent. Patients were identified at the Royal Free Hospital Renal Cancer Specialist Multi-Disciplinary Team Meeting (SMDT). Patients were recruited prospectively and were eligible to participate if they had a diagnosis of a T1 solid renal tumour made on standard of care imaging, were able to have an MRI and had not had a recent biopsy within the last three months. Histology from biopsy or surgical histology was available for all patients, performed as standard-of-care.

### VERDICT-MRI acquisition

VERDICT-MRI was performed on a 3T MRI system (Ingenia; Philips, Best, Netherlands), using a 16-channel body coil (SENSE XL Torso, Philips, Best, Netherlands), using a pulsed gradient spin-echo (PGSE) sequence with echo-planar imaging (EPI) readout in the coronal imaging plane. The acquisition was informed by the optimised protocol for prostate^38^, with additional measurements to obtain rich datasets for analysis and emphasis placed on lower *b*-values to reflect the vascularity of renal microstructure. The imaging parameters were as follows: repetition time (TR), 2000–3349 ms; field of view, 220 × 200 mm; voxel size, 1.25 × 1.25 × 5 mm; no interslice gap; acquisition matrix, 176 ×176. The VERDICT acquisition protocol for kidney is: *b* = [70, 90, 150, 500, 1000, 1500, 2000, 2200, 2500] s/mm^2^; δ = [4.8, 4.8, 4.8, 12.0, 12.0, 26.3, 16.8, 16.8, 21.4] ms; Δ = [27.0, 27.0, 27.0, 34.0, 34.0, 47.0, 37.5, 37.5, 43.5] ms. For each of the nine combinations of *b*/δ/Δ, we used the minimum possible echo time (TE), giving TEs of 54–87 ms. For each *b*-value, images were acquired in three orthogonal directions which were then spherically-averaged, and we acquired a separate image for each TE, resulting in 18 image volumes each with 14 slices. The acquisition time was approximately 46 min.

### Image analysis

The preprocessing pipeline included denoising using Marchenko–Pastur Principal Component Analysis (MP-PCA)^39^ (MrTrix3 ‘dwidenoise’^40^), and correction for Gibbs ringing^41^. We applied mutual-information rigid and affine registration^42^ and divided DW-MRI volumes by their matched *b* = 0 for normalisation. Regions of interest (ROIs) for tumour, simple cysts and normal renal parenchyma were drawn manually in ITKSnap v4.0 using co-registered T2-weighted images by a radiologist in-training with seven years’ experience in abdominal imaging^43^. Histological subtype and tumour WHO/ISUP (World Health Organisation/International Society of Urological Pathology) grade was determined from either lesion biopsy or surgery and compared to imaging tumour analysis. 10 unpaired surgical histological samples were of the main tumour types (ccRCC, pRCC, chRCC and oncocytoma) were stained with haematoxylin-and-eosin (H&E) and CD34 (a vascular endothelial marker) immunohistochemistry. Slides were scanned and the digital images analysed using the software QuPath^44^. Cell density and vascularity were used as histological validation of the VERDICT model.

### VERDICT model

The VERDICT model for kidney has three compartments that characterise the diffusion of water molecules in the intracellular (IC), vascular (VASC) and extracellular-extravascular space (EES) in tumours, with no exchange between the components^20,21^.

The total normalised signal is:

1 $${S}_{0}={f}_{{VASC}}{S}_{{VASC}}\left({d}_{{VASC}},b\right)+{f}_{{IC}}{S}_{{IC}}\left({d}_{{IC}},R,b,\Delta ,\delta \right)+{f}_{{EES}}{S}_{{EES}}\left({d}_{{EES}},b\right)$$

where ${f}_{i}$ is the volume fraction and ${S}_{i}$ is the normalised signal from water molecules in population $i$, where $i=$IC, VASC or EES. The vascular signal fraction, ${f}_{{VASC}}$, is computed as $1-{f}_{{IC}}-{f}_{{EES}}$, since $0\le {f}_{i}\le 1$ and ${\sum }_{i=0}^{1}{f}_{i}=1$, and ${S}_{0}$ is the signal with no diffusion weighting. Here $b$ is the *b*-value, $\Delta$ is the gradient pulse separation and $\delta$ is the gradient pulse duration.

To represent renal tissue, the IC compartment was modelled as an impermeable sphere of radius $R$ with IC diffusivity fixed at ${d}_{{IC}}=2$µm^2^/ms and the EES compartment as Gaussian free diffusion with diffusivity ${d}_{{EES}}=2$µm^2^/ms. The vascular compartment was randomly-oriented sticks with pseudo-diffusivity ${d}_{{VASC}}=50$µm^2^/ms to reflect the high vascularity of kidney tissue (see Supplementary Fig. 1). Three free parameters were estimated: ${f}_{{IC}}$, ${f}_{{EES}}$ and $R$ within biophysically-realistic parameter ranges: [0, 1], [0, 1], [0, 15 µm].

### Model fitting

The renal VERDICT model is fit to the data using self-supervised deep learning, previously used in ref. ^27^ with the prostate model. These techniques remove the need for training data, instead extracting labels from the input data itself. They work to estimate the model parameters and reconstruct the signal, then calculate the training loss as the MSE between the predicted signal, S^, and the input signal S^45^ to find the optimal estimates. A fully connected neural network with three hidden layers, each with 18 neurons (i.e. the number of image volumes, a spherically-averaged *b*-value image with a corresponding b_0_), was implemented using *PyTorch* 1.12.1. The output layer is fed into the VERDICT model equation to generate the predicted signal S^. We used the ADAM optimiser, early stopping to mitigate overfitting, and a learning rate of 0.0001. We used dropout of *p* = 0.5 and constrained the parameter values to the ranges above using the *PyTorch* clamp function. Training and inference are performed simultaneously and took about 50 s for each masked dMRI data set.

### Protocol optimisation

The protocol mentioned above provides rich DW datasets useful for model development and analysis, however the acquisition time of 46 min is not clinically feasible. We develop a dual-network deep learning approach based on similar work in literature^29,30,46^ to select the most informative subset from the full protocol of nine *b*-values.

Features were selected from 27 measurements from the DW images acquired along the three orthogonal directions per *b*-value. The non-DW measurements were excluded to prevent them from being chosen, as they would be included in the protocol with their matched DW. Each DW dataset was normalised and fed into the first network for feature selection. Each dataset was normalised and input into a Feature Scoring Network – a fully connected model with 27 input nodes, one hidden layer with 64 units (using ReLU activation and batch normalisation), and a 27-node output layer with a sigmoid activation. This network produced importance scores in the range [0,1] for each measurement of a dataset. The 12 highest-scoring features were selected for each dataset and passed into a Prediction Network with 12 input nodes, a hidden layer of 64 units (ReLU activation), and an output layer of 27 nodes, which aimed to reconstruct the full measurement set from the chosen subset of 12 measurements.

**Fig. 6:**
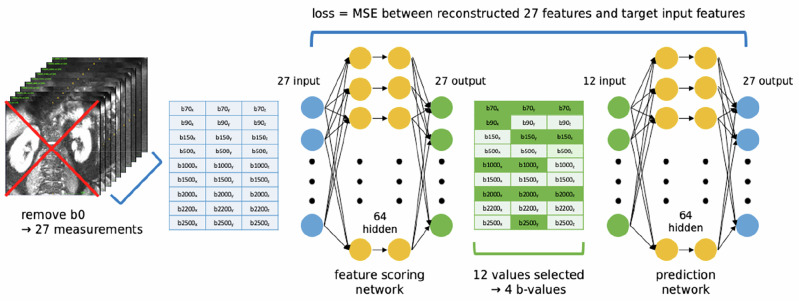
Schematic outlining the protocol optimisation dual-network strategy. We first remove the non-DW images from the image volumes, resulting in 27 measurements per patient. Our feature scoring network takes in the 27 measurements (27 input nodes), has hidden layers with 64 nodes and outputs 27 scores for the measurements. From these, the 12 measurements with the highest scores are selected. These are then used to reconstruct the target initial 27 measurements in the predictor network, and the loss is computed as the MSE between the output and the target. We evaluate on our full patient dataset, choosing b = 70, 150, 1000, 2000 as our final protocol. This gives a reduction in acquisition time of 32 min.

The two networks were trained in tandem for 100 epochs and the loss was computed as the MSE between the reconstructed full measurement and ground truth target values (the input 27 measurements). A learning rate of 1e^−5^ was used, with the ADAM optimiser and dropout to prevent overfitting. Upon training completion, we extracted the final optimal feature set by computing the average importance scores across all subjects and extracting a final protocol of 4 *b*-values (12 measurements in 3 directions). The method is outlined in Fig. 6.

### Statistical analysis

Data were analysed using Python 3.11 (SciPy 1.15.0^47^) and statistical tests performed were Wilcoxon’s signed-rank tests with errors reported as standard deviation, unless stated otherwise. *p-*values are summarised in figures as: <0.0001, ****; 0.0001–0.001, ***; 0.001–0.01, **; 0.01–0.05, *.

## Supplementary information

Editable_checklist (1).

npj_supplementary_amended.

## Data Availability

The datasets generated and/or analysed during the current study are not publicly available as participants in the pilot study consented to data use for research purposes, but not to public online sharing. Data are available from the corresponding author on reasonable request, in line with ethical guidelines.

## Code availability

The underlying code for this study [and training/validation datasets] is not publicly available but may be made available to qualified researchers on reasonable request from the corresponding author.
