# Supplementary material for: Dual deep learning approach for non-invasive renal tumour subtyping with VERDICT-MRI
Source: Npj Imaging. 2026 Jan 6;4:2. doi: 10.1038/s44303-025-00135-6 (PMC12774943; doi:10.1038/s44303-025-00135-6)
Supplement: Supplementary file 1 — Editable_checklist (1). [file 44303_2025_135_MOESM1_ESM.docx]

**Supplementary Materials**


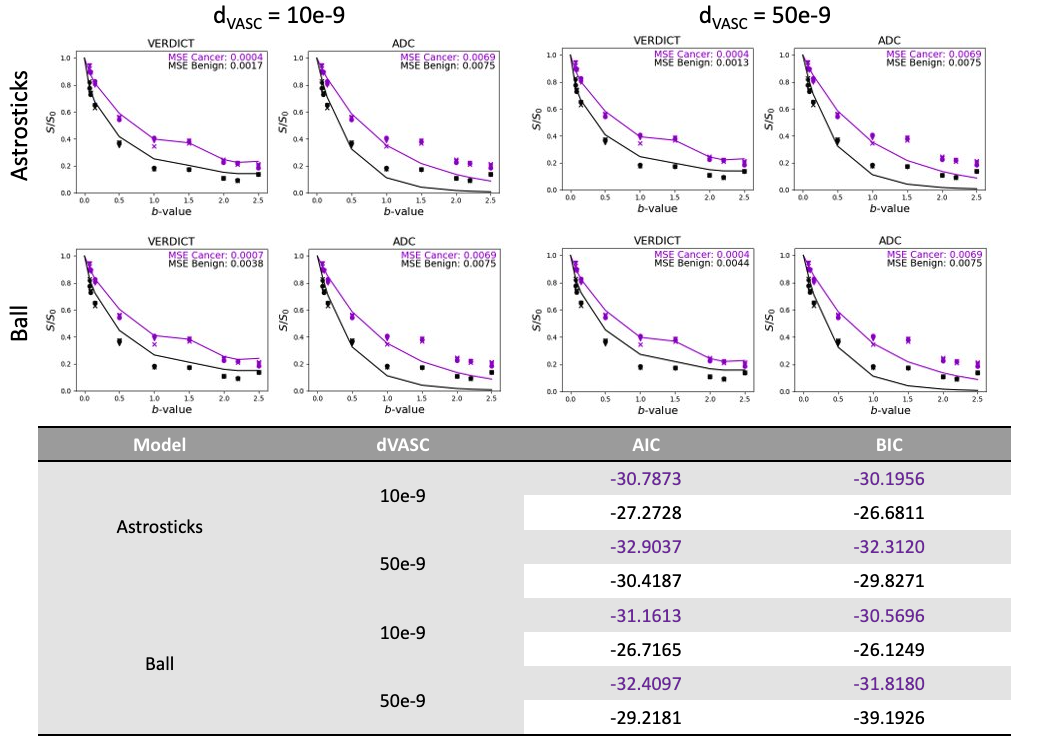


**Supplementary Fig. 1:** **Modelling analysis to develop a VERDICT model for renal tissue.**

We experimented with the vascular compartment, trialling both a ‘ball’ and ‘astrosticks’ geometry with d_VASC_ = 10m^2^/ms and d_VASC_ = 50m^2^/ms. We found that the ‘astrosticks’ compartment with d_VASC_ = 50m^2^/ms was the best fit to the DW data and had the lowest Akaike’s Information Criterion (AIC) and Bayesian Information Criterion (BIC), meaning they provide a better description of the data. The purple values in the table translate to the values in the cancerous regions, and the black values are for benign regions.


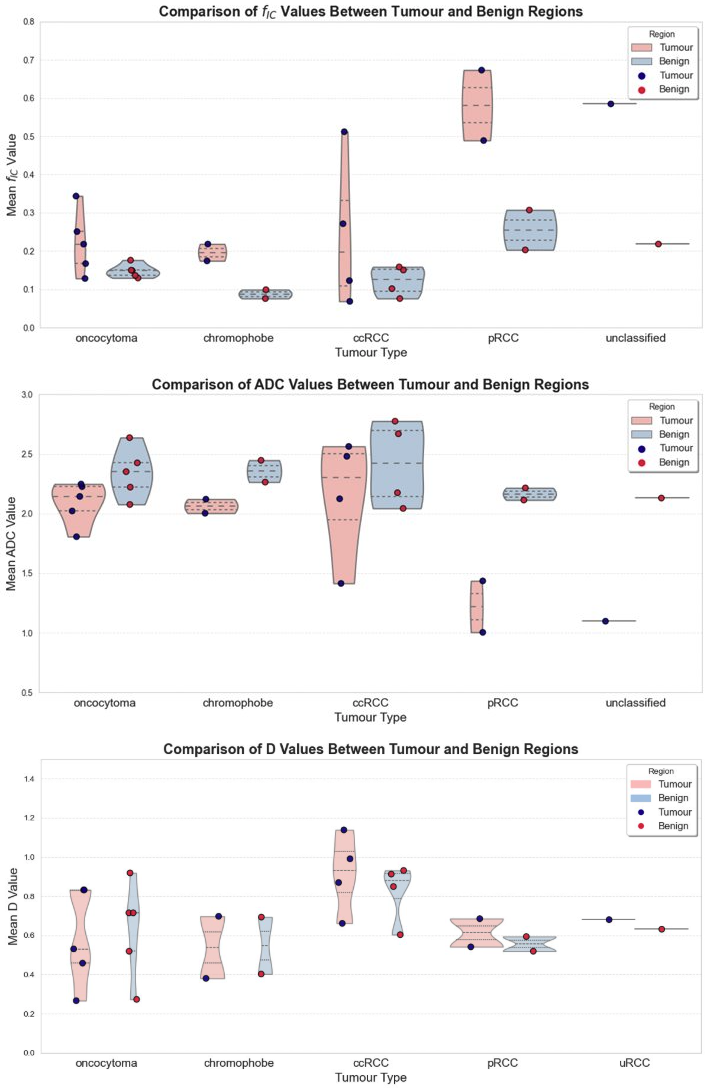


**Supplementary Fig. 2: Parameter Estimates in RCC Subtypes**

Violin plots showing (A) f_IC_ and (B) ADC and (C) IVIM D estimates in patient groups. We observe good separation between tumour and healthy tissue for chRCC, pRCC and uRCC with fIC and ADC, but not for the other groups. There is a large spread in tumour estimates for ccRCC, possibly due to the range of tumour grades in our cohort. The plots suggest that oncocytoma and chRCC have similar traits, which is supported by the histology in Fig. 1, whilst pRCC and uRCC have higher f_IC_/lower ADC.


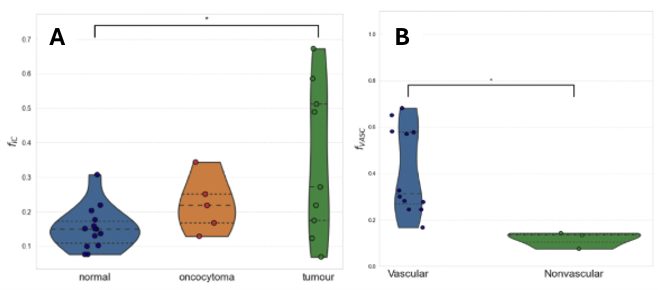


**Supplementary Fig. 3:** **Optimised Protocol Violin Plots**

Recreations of the VERDICT violin plots in Fig. 4, with the reduced protocol identified via the feature selection network. We observe that we achieve statistical significance with f_IC_ at p < 0.05 and the same statistical significance with the f_VASC_ as we obtained with the full protocol. This demonstrates that we do not lose significant microstructural information with our significantly shorter acquisition.
